# Prevalence and risk factors of pre-hypertension and hypertension among adults in Southeastern Iran: Findings from the baseline survey of the Zahedan adult cohort study

**DOI:** 10.1371/journal.pone.0295270

**Published:** 2023-12-07

**Authors:** Mojtaba Gholami Zare, Hassan Okati-Aliabad, Alireza Ansari-Moghaddam, Mahdi Mohammadi, Fariba Shahraki-Sanavi

**Affiliations:** 1 Department of Epidemiology, MSc Candidate of Epidemiology, School of Public Health, Zahedan University of Medical Sciences, Zahedan, Iran; 2 Health Promotion Research Center, Zahedan University of Medical Sciences, Zahedan, Iran; Shahid Beheshti University of Medical Sciences School of Medicine, ISLAMIC REPUBLIC OF IRAN

## Abstract

Hypertension (HTN) stands as the leading modifiable risk factor for cardiovascular disease(CVD) and premature death globally. Understanding its prevalence and risk factors is essential for effective prevention and management of HTN. This study aimed to investigate the prevalence of Pre-hypertension (pre-HTN), HTN, and its risk factors in adults participating in the Zahedan adult cohort study (ZACS). This cross-sectional study used the baseline data of the ZACS. Ordinal logistic regression analysis was used to estimate crude and adjusted odds ratios (ORs) with 95% confidence intervals (CIs) for potential risk factors. Among the 10,016 participants in this study, 60.89% were women, with an average age of 50.44 ± 9.18 years. The prevalence of pre-HTN and HTN was 42.03% (men 45.44%, women 39.84%) and 18.47% (men 21.09%, women 16.79%), respectively. Being male, older age, having higher socioeconomic status (SES), being overweight and obese, having a family history of HTN, comorbidities such as diabetes and CVD, as well as abnormal blood lipid levels (triglycerides and HDL cholesterol) were the most significant predictors of pre-HTN and HTN. These findings highlight that more than half of the participants in this study exhibit pre-HTN or HTN, placing them at risk for CVD and stroke. Implementing comprehensive preventive strategies tailored to these identified risk factors is imperative to alleviate the disease burden, enhance disease management, and improve HTN treatment and control.

## Introduction

NCDs are the most serious public health concern in the 21st century, threatening societies’ social and economic development, responsible for 71% (41 million people) of all deaths worldwide [1,2]. Key modifiable risk factors for NCDs include hypertension (HTN), diabetes, obesity, and dyslipidemia [3]. HTN, in particular, is considered one of the main risk factors of NCDs worldwide [4]. It is the leading cause of myocardial infarction, heart failure, stroke, chronic kidney disease, decreased cognitive function, and an increased risk of death from CVD [5,6]. Notably, HTN is also the most important known risk factor for disease burden in all age groups, especially those over 50 years of age, surpassing tobacco and malnutrition [7,8].

According to the Global Burden of Disease (GBD), HTN accounted for approximately 10.8 million deaths in 2019, equating to 9.3% disability-adjusted life years (DALYs). Furthermore, It is predicted that the global number of hypertensive patients will reach 1.56 billion by 2025 [8]. The Seventh Report of the National Committee for the Prevention, Diagnosis, Evaluation, and Treatment of Blood Pressure (JNC-7) reported that individuals with pre-HTN are at greater risk for CVD and death than individuals with normal blood pressure. Pre-HTN is associated with a 1.7-fold increase in coronary artery disease and a 3.5-fold rise in myocardial infarction [9,10].

Importantly, the burden of HTN has shifted from developed to developing countries. Increased life expectancy, rapid urbanization, unhealthy diets, and lifestyle changes have increased CVD and its risk factors, such as HTN, in low- and middle-income countries during the last century [11]. Projections indicate that by 2025, the global prevalence of HTN will affect 29.2% of the total population and 60% of adults. The total percentage of DALY related to HTN increased from 6% to more than 10% between 1990 and 2019, demonstrating the importance and necessity of basic measures in this field [8]. It’s essential to note that more than 80% of NCD victims live in low- and middle-income communities [12]. In the Middle East and North Africa, there has been a significant increase in the prevalence of HTN over the past three decades. Prevalence rates for pre-HTN and HTN were 33% and 26%, respectively [13].

The economic burden of NCDs, including HTN, is much higher in low- and middle-income countries due to various factors such as health costs, poor governance, inefficient healthcare systems, and a focus on treatment rather than prevention. Individuals, families, healthcare systems, and entire countries in low- and middle-income countries face significant economic pressures due to HTN [14,15]. In these countries, the monthly cost of treating HTN is around $ 22 per person [14]. Therefore, recognizing and controlling HTN is the most cost-effective way to prevent premature CVD in different populations, especially in low- and middle-income countries [16].

Iran is in a transitional phase marked by an aging population and a rising burden of NCDs [17]. In Iran, HTN is a major risk factor for NCDs, particularly CVDs. In 2019, HTN contributed to approximately 30.6% of NCD-related deaths and 13.8% of disability-adjusted life years (DALYs) in Iran. However, awareness of HTN and pre-hypertension remains low [18]. Meta-analysis studies in Iran reveal that the prevalence of pre-HTN and HTN is 31.6 and 20.4 percent, respectively, which is relatively high and considered a public health concern in the country. The increase in HTN cases in Iran is attributed to the rapid social and economic development associated with adopting a sedentary and Western lifestyle [19,20].

Despite cross-sectional studies in Iran on HTN prevalence, comprehensive investigations on its risk factors, particularly in southern Iran, have been relatively scarce. Therefore, this study aims to shed light on the prevalence and risk factors of this significant CVD risk factor in the Zahedan adult cohort study population in southeast Iran.

## Materials and methods

### Study design

This cross-sectional study used the baseline data of the ZACS in the Southeast of Iran. The ZACS was part of the prospective epidemiological research studies of Iran (PERSIAN) and was conducted on 10,016 individuals aged 35–70 years. Recruitment and data collection occurred between October 2015 and January 2019 in Zahedan. This study’s rationale, objectives, and design have been previously published [21]. The inclusion criteria were Iranian citizenship, age 35–70 years at the time of the baseline survey, accommodation for at least 9 months in Zahedan for residents, and at least 1 year of residency for immigrants from other areas. Data were collected after obtaining written informed consent from the participants. People who did not comply with study requirements, or had a severe physical or mental illness and could not answer the questionnaires or refer to the cohort center, were excluded from the study. The reference population was selected by a multi-stage random sampling method. In the first stage, the regions of Zahedan city were divided based on SES. In the next step, three regions, low, middle, and high SES, were randomly selected, and then all eligible residents in these regions were invited to participate in the study. Finally, 10,016 people were included in the study. For this study, the data was provided to the research team by the supervisor of the ZACS in a coded and anonymous form, and the authors did not have access to the participants’ identification information. The Ethics Committee of Zahedan University of Medical Sciences approved the study (IR.ZAUMS.REC.1400.105).

### Measurements

Trained personnel used valid questionnaires to collect information about socio-demographic characteristics, substance use, medical history, family history, SES, and physical activity. Anthropometric indices, blood pressure measurements, and blood lipid tests were conducted as part of the assessment. The participants were classified into four groups: 35–44, 45–54, 55–64, and over 64 years old to determine the prevalence of pre-HTN and HTN in different age groups. To determine participants’ socioeconomic status (SES), this study employed the same asset-based wealth index method as in the previous study [22]. The wealth score index was calculated using multiple correspondence analysis (MCA) of various variables, including access to facilities, travel status, home ownership, number of bedrooms, monthly household income, domestic and international trips per year, and number of books read. Multiple correspondence analysis (MCA) is a widely used technique to analyze categorical data and aims to reduce large sets of variables into smaller sets of components that summarize the information contained in the data. MCA can be regarded as an adaptation to the categorical data of principal component analysis (PCA) [23].

Participants’ physical activity was classified into three classes (low, <3.0 METs; moderate, 3.0–5.9 METs; high ≥6.0) based on the 24-hour activity level and Metabolic Equivalent Task (MET) index [24]. We calculated the body mass index (BMI) of the participants by dividing their weight (kg) by their height (m2) and categorizing them accordingly, BMI < 18.5 kg / m2 as underweight, 18.5–24.9 kg / m2 as normal, 25–29.9 kg / m2 as overweight, and more than 30 as obese. Waist circumference (WC) > 102 cm in men and > 88 cm in women was considered abdominal obesity [25]. Triglyceride(TG) ≥150 mg/dl, low-density lipoprotein (LDL)≥ 130 mg/dl, and high-density lipoprotein (HDL) ≤40 mg/dl were considered abnormal blood lipids [25,26]. Diabetes mellitus was defined as fasting blood sugar (FBS) ≥126 mg/dl or a history of taking diabetes medications. Current cigarette smokers were defined as individuals who had smoked at least 100 cigarettes in their lifetime and currently smoked either every day or on some days [27].

Blood pressure was measured using a standard calibrated sphygmomanometer (Reister Model, Germany) with an appropriate-size cuff. Participants refrained from smoking, strenuous activity, heavy foods, coffee, alcohol, drugs, or stimulant beverages for at least 30 minutes before measurement. After 5 minutes of seated rest, blood pressure was recorded in two stages, 10 minutes apart, for both the right and left arms. The average of these measurements determined systolic and diastolic blood pressure. HTN was defined per JNC-7 criteria (26) as systolic blood pressure (SBP) ≥ 140 mm Hg, diastolic blood pressure (DBP) ≥ 90 mm Hg, or use of HTN medication. Pre-hypertension (Pre-HTN) was defined as SBP of 120–139 mm Hg or DBP of 80–89 mm Hg in individuals not previously treated for hypertension [28].

### Statistical analysis

All statistical analyses were performed using STATA v.14.2 (Stata Corp. 2015 College Station, TX: Stata Corp LP) software. For descriptive analysis, mean and standard deviation (SD) were used for quantitative data, and frequency distribution for qualitative data. The partial proportional odds model for the ordinal response variable was used for multi-factor analysis for all significant variables in uni-factor analysis to evaluate the relationship between independent and response variables. Crude odds ratio (COR) and Adjusted odds ratio (AOR) with a 95% confidence interval (CI) were revealed. A significant value at P <0.05 was considered.

## Results

### Characteristics of study participants

A total of 10,016 participants with a mean age of 50.4±9.2 years participated in this study. Most individuals were female (60.9%), married (88.3%), and house owners (74.1%). About half of the participants were of Sistani ethnicity (50.3%) and most of them had elementary or secondary education (64.6%) with low or middle socio-economic status (72.2%). Some of the participants were cigarette smokers (16.4%), alcohol users (2.4%), and drug users (19.0%). Low physical activity and depression were 46.6% and 15.8%, respectively. Participants reported a history of CVD (9.1%), diabetes (18.9%), and stroke (1.6%). Most of the people were overweight or obese (66.3%) and there were abnormal WC (56.7%), abnormal TG levels (34.7%), abnormal LDL levels (17.0%), and abnormal HDL levels (32.7%) (Table 1).

**Table 1:** Demographic, anthropometric, and clinical characteristics of the participants.

Variables	Total no (%)	Normal, no (%)	Pre-hypertension, no (%)	Hypertension, no (%)
**Total**	10016 (100%)	3956 (39.50%)	4210 (42.03%)	1850 (18.47%)
**Sex** Male Female	3917 (39.11%) 6099 (60.89%)	1311(33.47%) 2645(43.37%)	1780 (45.44%) 2430 (39.84%)	826 (21.09%) 1024 (16.79%)
**Age (Year)** 35–44 45–54 55–64 >64	3093 (30.89%) 3316 (33.10%) 3070 (30.65%) 537 (5.36%)	1800 (58.19%) 1315 (39.65%) 743 (24.20%) 98 (18.25%)	1080 (34.92%) 1445 (43.58%) 1455 (47.39%) 230 (42.83%)	213 (6.89%) 556 (16.77%) 872 (28.40%) 209 (38.92%)
**Education** University Secondary Elementary Illiterate	1236 (12.34%) 3943 (39.37%) 2532 (25.28%) 2305 (23.01%)	476 (38.51%) 1657 (42.02%) 1062 (41.94%) 761 (33.02%)	532 (43.04%) 1612 (40.88%) 1079 (42.61%) 987 (42.82%)	228 (18.45%) 674 (17.09%) 391 (15.44%) 557 (24.16%)
**Marital status** Single Married Other (widowed, divorced)	174 (1.74%) 8841 (88.27%) 1001 (9.99%)	97 (55.75%) 3483 (39.40%) 376 (37.56%)	55 (31.61%) 3723 (42.11%) 432 (43.16%)	22 (12.64%) 1635 (18.49%) 193 (19.28%)
**Ethnicity** Sistani Baluch Other	5039 (50.31%) 2865 (28.60%) 2112 (21.09%)	1941 (38.52%) 1194 (41.68%) 821 (38.87%)	2113 (41.93%) 1205 (42.06%) 892 (42.23%)	985 (19.55%) 466 (16.27%) 399 (18.89%)
**Housing status** Rent Owner	2590 (25.86%) 7426 (74.14%)	1261 (48.69%) 2695 (36.29%)	1009 (38.96%) 3201 (43.11%)	320 (12.36%) 1530 (20.60%)
**Socio-economic status** Low Middle High	2593 (25.89%) 4634 (46.27%) 2789 (27.85%)	1120 (43.19%) 1788 (38.58%) 1048 (37.58%)	1043 (40.22%) 1990 (42.94%) 1177 (42.20%)	430 (16.58%) 856 (18.47%) 564 (20.22%)
**BMI** Normal Underweight Overweight Obese	2641 (26.36%) 727 (7.28%) 3828 (38.24%) 2815 (28.12%)	1259 (47.67%) 474 (65.19%) 1413 (36.91%) 808 (28.70%)	1043 (39.49%) 195 (26.83%) 1645 (42.97%) 1325 (47.07%)	339 (12.84%) 58 (7.98%) 770 (20.11%) 682 (24.23%)
**Waist circumference** Normal (Men<102, Women<88) Abnormal (Men>102, Women>88)	4330 (43.28%) 5675 (56.72%)	1969 (45.47%) 1984 (34.96%)	1695 (39.15%) 2510 (44.23%)	666 (15.38%) 1181 (20.81%)
**Physical activity** Low Moderate High	4672 (46.65%) 4639 (46.32%) 705 (7.04%)	1721 (36.84%) 1924 (41.47%) 311 (44.11%)	2007 (42.96%) 1905 (41.06%) 298 (42.27%)	944 (20.21%) 810 (17.46%) 96 (13.62%)
**History of CVD** No Yes	9102 (90.87%) 914 (9.13%)	3751 (41.21%) 205 (22.43%)	3791 (41.65%) 419 (45.84%)	1560 (17.14%) 290 (31.73%)
**History of diabetes** No Yes	8119 (81.06%) 1897 (18.94%)	3492 (43.01%) 464 (24.46%)	3305 (40.71%) 905 (47.71%)	1322 (16.28%) 528 (27.83%)
**Family history of HTN** No Yes	4868 (48.61%) 5147 (51.39%)	2000 (41.08%) 1956 (38.00%)	2054 (42.19%) 2156 (41.89%)	814 (16.72%) 1035 (20.11%)
**Depression** No Yes	8438 (84.25%) 1578 (15.75%)	3332 (39.49%) 624 (39.54%)	3527 (41.80%) 683 (43.28%)	1579 (18.71%) 271 (17.17%)
**History of Stroke** No Yes	9860 (98.44%) 156 (1.56%)	3918 (39.74%) 38 (24.36%)	4141 (42.00%) 69 (42.23%)	1801 (18.27%) 49 (31.41%)
**TG level** Normal Abnormal	6498 (65.27%) 3457 (34.73%)	2798 (43.06%) 1135 (32.83%)	2658 (40.90%) 1523 (44.06%)	1042 (16.04%) 799 (23.11%)
**LDL level** Normal Abnormal	8229 (83.00%) 1686 (17.00%)	3314 (40.27%) 610 (36.18%)	3434 (41.73%) 732 (43.42%)	1481 (18.00%) 344 (20.40%)
**HDL level** Normal Abnormal	6712 (67.30%) 3261 (32.70%)	2794 (41.63%) 1144 (35.08%)	2784 (41.48%) 1405 (43.08%)	1134 (16.90%) 712 (21.83%)
**Drug use** No Yes	8112 (81.00%) 1903 (19.00%)	3208 (39.55%) 748 (39.31%)	3421 (42.17%) 789 (41.46%)	1483 (18.28%) 366 (19.23%)
**Alcohol Use** No Yes	9779 (97.64%) 236 (2.36%)	3851 (39.38%) 105 (44.49%)	4112 (42.05%) 98 (41.53%)	1816 (18.57%) 33 (13.98%)
**Smoking** No Yes	8375 (83.62%) 1640 (16.38%)	3300 (39.40%) 656 (40.00%)	3507 (41.87%) 703 (42.87%)	1568 (18.72%) 281 (17.13%)

### Prevalence and risk factors of pre-HTN and HTN

The prevalence of pre-HTN and HTN was 42.03% (men 45.44%, women 39.84%) and 18.47% (men 21.09%, women 16.79%), respectively (Table 2). The crude odds of pre-HTN/HTN compared to the normal status, as well as the odds of HTN compared to pre-HTN/normal status in terms of associated factors, are illustrated in Table 2.

**Table 2:** Crude OR (95% CI) of factors associated with pre-hypertension and hypertension.

HTN Variable	(Pre-HTN, HTN) vs. Normal		HTN vs. (Pre-HTN, normal)	
	Crude OR (95%CI)	P	Crude OR (95%CI)	P
**Sex** Female Male	1.00 1.52 (1.40, 1.65)	<0.001	1.00 1.32 (1.19, 1.46)	<0.001
**Age (Year)** 35–44 45–54 55–64 >64	1.00 2.26 (2.04, 2.50) 4.33(3.89, 4.82) 6.19 (4.92, 7.79)	<0.001 <0.001 <0.001	1.00 2.75 (2.34, 3.23) 5.15(4.42, 6.00) 8.28 (6.66, 10.28)	<0.001 <0.001 <0.001
**Education** University Secondary Elementary Illiterate	1.00 0.86 (0.75, 0.98) 0.86 (0.75, 0.99) 1.27 (1.10, 1.46)	0.035 0.068 <0.001	1.00 0.91 (0.77, 1.07) 0.80 (0.67, 0.96) 1.40 (1.18, 1.67)	0.035 0.001 <0.001
**Marital status** Single Married Other(widowed, divorced)	1.00 1.93 (1.43, 2.62) 2.09 (1.51, 2.89)	<0.001 <0.001	1.00 1.93 (1.43, 2.62) 2.09 (1.51, 2.89)	<0.001 <0.001
**Ethnicity** Other Sistani Baluch	1.00 1.01 (0.91, 1.12) 0.88 (0.79, 0.99)	0.619 0.012	1.00 1.01 (0.91, 1.12) 0.88 (0.79, 0.99)	0.619 0.012
**Housing status** Rent Owner	1.00 1.66 (1.52, 1.82)	<0.001	1.00 1.66 (1.52, 1.82)	<0.001
**SES** Low Middle High	1.00 1.21 (1.09, 1.33) 1.26 (1.13, 1.40)	<0.001 <0.001	1.00 1.21 (1.09, 1.33) 1.26 (1.13, 1.40)	<0.001 <0.001
**BMI** Normal Underweight Overweight Obese	1.00 0.42 (0.34, 0.53) 1.64 (1.49, 1.81) 2.39 (2.14, 2.67)	<0.001 <0.001 <0.001	1.00 0.42 (0.34, 0.53) 1.64 (1.49, 1.81) 2.39 (2.14, 2.67)	<0.001 <0.001 <0.001
**HTN** **Variable**	**(Pre-HTN, HTN) vs. Normal**		**HTN vs. (Pre-HTN, normal)**	
	**Crude OR (95%CI)**	**P**	**Crude OR (95%CI)**	**P**
**WC** Normal (Men<102, Women<88) Abnormal (Men>102, Women>88)	1.00 1.55 (1.43, 1.68)	<0.001	1.00 1.55 (1.43, 1.68)	<0.001
**Physical activity** Low Moderate High	1.00 0.82 (0.75, 0.89) 0.73 (0.62, 0.86)	<0.001 <0.001	1.00 0.82 (0.75, 0.89) 0.73 (0.62, 0.86)	<0.001 <0.001
**History of CVD** No Yes	1.00 2.42 (2.06, 2.84)	<0.001	1.00 2.42 (2.06, 2.84)	<0.001
**History of diabetes** No Yes	1.00 2.33 (2.08, 2.61)	<0.001	1.00 1.98 (1.76, 2.22)	<0.001
**Family history HTN** No Yes	1.00 1.13 (1.05, 1.23)	<0.001	1.00 1.13 (1.05, 1.23)	<0.001
**History of depression** No Yes	1.00 0.99 (0.89, 1.11)	0.507	1.00 0.99 (0.89, 1.11)	0.507
**History of Stroke** No Yes	1.00 2.04 (1.41, 2.95)	<0.001	1.00 2.04 (1.41, 2.95)	<0.001
**TG level** Normal Abnormal	1.00 1.54 (1.41, 1.68)	<0.001	1.00 1.54 (1.41, 1.68)	<0.001
**LDL level** Normal Abnormal	1.00 1.18 (1.07, 1.32)	0.001	1.00 1.18 (1.07, 1.32)	0.001
**HDL level** Normal Abnormal	1.00 1.31 (1.20, 1.43)	<0.001	1.00 1.31 (1.20, 1.43)	<0.001
**Drug use** No Yes	1.00 1.02 (0.92, 1.12)	0. 570	1.00 1.02 (0.92, 1.12)	0. 570
**Alcohol Use** No Yes	1.00 0.81 (0.62, 1.05)	0. 110	1.00 0.81 (0.62, 1.05)	0. 110
**Smoking** No Yes	1.00 0.94 (0.84, 1.04)	0. 185	1.00 0.94 (0.84, 1.04)	0. 185

The Odds of pre-HTN/HTN were significantly higher in married (OR = 1.9, P<0.001) and widowed/divorced (OR = 2.09, P<0.001) compared to single participants, middle or high SES (OR = 1.2 P<0.001) compared to low SES, and house owners (OR = 1.66, P<0.001) compared to those who rented a house. In contrast to Baluch ethnicity (OR = 0.88, P = 0.012), male participants (OR = 1.5, P<0.001) were more likely to have pre-HTN/HTN. The odds of pre-HTN/HTN increased in older individuals. Compared to people aged 35–44 years, the odds of Pre-HTN/HTN increased from 2.3 in people aged 45–54 years (P<0.001) to 6.2 in those older than 64 years (P<0.001). Compared to university graduates, the odds of pre-HTN/HTN was 1.3 (P<0.001) in illiterate and 0.86 (P = 0.035) in secondary education individuals. For all factors mentioned, the odds of HTN against pre-HTN/normal were similar (Table 2).

In contrast to underweight participants (OR = 0.4, P<0.001), overweight (Or = 1.6, P<0.001) and obese participants (OR = 2.4, P<0.001) were more likely than individuals with normal status to have pre-HTN/HTN. Abnormal WC increased the odds of pre-HTN/HTN (OR = 1.5, P<0.001). People with moderate (OR = 0.8, P<0.001) and high physical activity (OR = 0.7, P<0.001) were less likely to have pre-HTN/HTN compared to those with low physical activity (Table 2).

The Odds of pre-HTN/HTN were significantly higher in people with a history of CVD (OR = 2.4, P<0.001), history of diabetes (OR = 2.3 P<0.001), history of HTN (OR = 1.1, P<0.001), and history of stroke (OR = 2, P<0.001).

People with an abnormal TG level (OR = 1.5, P<0.001), abnormal LDL level (OR = 1.2, P = 0.001), and abnormal HDL level (OR = 1.3, P<0.001) were more likely to have pre-HTN/HTN. The same results were found for comparing HTN with pre-HTN/ normal status (Table 2).

### Factors associated with pre-HTN and HTN in multi-factors analysis

Adjusted odds of pre-HTN/HTN against normal status and also odds of HTN against pre-HTN/normal status in terms of associated factors are illustrated in Table 3.

**Table 3:** Factors associated with pre-hypertension and hypertension: Ordinal logistic regression.

HTN Variable	(Pre-HTN, HTN) vs. Normal	HTN vs. (Pre-HTN, normal)
	Adjusted OR (95%CI)	P	Adjusted OR (95%CI)	P
**Sex** Female Male	1.00 2.08 (1.83, 2.36)	<0.001	1.00 1.48 (1.27, 1.73)	<0.001
**Age (Year)** 35–44 45–54 55–64 >64	1.00 1.99 (1.79, 2.22) 3.66 (3.24, 4.14) 5.56 (4.33, 7.14)	<0.001 <0.001 <0.001	1.00 2.44 (2.06, 2.88) 4.51 (3.82, 5.32) 8.02 (6.31, 10.20)	<0.001 <0.001 <0.001
**Education** University Secondary Elementary Illiterate	1.00 1.00 (0.83, 1.20) 0.97 (0.79, 1.19) 1.28 (1.03, 1.59)	0.551 0.949 0.054	1.00 1.00 (0.83, 1.20) 0.97 (0.79, 1.19) 1.28 (1.03, 1.59)	0.551 0.949 0.054
**SES** Low Middle High	1.00 1.05 (0.91, 1.21) 1.21 (1.02, 1.43)	0.266 0.019	1.00 1.05 (0.91, 1.21) 1.21 (1.02, 1.43)	0.266 0.019
**BMI** Normal Underweight Overweight Obese	1.00 0.45 (0.36, 0.58) 1.51 (1.34, 1.70) 2.37 (2.04, 2.75)	<0.001 <0.001 <0.001	1.00 0.45 (0.36, 0.58) 1.51 (1.34, 1.70) 2.37 (2.04, 2.75)	<0.001 <0.001 <0.001
**WC** Normal (Men<102, Women<88) Abnormal (Men>102, Women>88)	1.00 1.29 (1.13, 1.49)	<0.001	1.00 1.06 (0.90, 1.26)	0.409
**History of CVD** No Yes	1.00 1.25 (1.07, 1.46)	0.001	1.00 1.25 (1.07, 1.46)	0.001
**History of diabetes** No Yes	1.00 1.38 (1.22, 1.57)	<0.001	1.00 1.38 (1.22, 1.57)	<0.001
**Family history HTN** No Yes	1.00 1.30 (1.19, 1.42)	<0.001	1.00 1.30 (1.19, 1.42)	<0.001
**TG level** Normal Abnormal	1.00 1.14 (1.03, 1.25)	0.007	1.00 1.14 (1.03, 1.25)	0.007
**HDL level** Normal Abnormal	1.00 1.06 (0.96, 1.16)	0.293	1.00 1.17 (1.04, 1.32)	0.003

Male individuals were more likely to have pre-HTN/HTN (OR = 2.1, P<0.001) and also HTN (OR = 1.5, P<0.001). The odds of pre-HTN/HTN and HTN increased for older people. Compared to people aged 35–44 years, the odds of pre-HTN/HTN increased from 2 (P<0.001) in people aged 35–44 to 5.6 (P<0.001) in individuals older than 64 years. The odds of HTN were almost twice in every next 10-year period.

Illiterate people were more likely to have pre-HTN/HTN (OR = 1.3, P = 0.05) than university graduates. Compared to people with normal status, pre-HTN/HTN was more prevalent in overweight (OR = 1.5, P<0.001) and obese people (OR = 2.4, P<0.001) in contrast to underweight individuals (OR = 0.4, P<0.001). People with high SES were more likely to have pre-HTN/HTN than those with low SES (OR = 1.2, P = 0.019). For all factors mentioned, the same results were obtained for the comparison of HTN against pre-HTN/normal status (Table 3).

Although the odds of pre-HTN/HTN were 1.3 (P<0.001) times more in people with abnormal WC, the odds of HTN were not significantly different between individuals with normal and abnormal WC (OR = 1.06, P = 0.409). Odds of pre-HTN/HTN increased in people with a history of CVD (OR = 1.2, P = 0.001), and a history of diabetes (OR = 1.4, P<0.001). The odds of HTN were similar for people with a history of the same diseases (Table 3).

Abnormal TG level increased the odds of pre-HTN/HTN (OR = 1.14, P = 0.007) which was the same for the odds of HTN. Although abnormal HDL increased the odds of HTN (OR = 1.17, P = 0.003), the odds of pre-HTN/HTN were not significantly different between people with normal and abnormal HDL (OR = 1.06, P = 0.0293) (Table 3).

## Discussion

This study provides an estimate of the prevalence of pre-HTN and HTN and their associated risk factors in the adult population of Zahedan. This study indicates that over 50% of Zahedan’s adult population has pre-HTN and HTN, with predictors including gender, age, education, SES, overweight and obesity, comorbidity of CVD and diabetes, family history of HTN, and abnormal blood lipids.

The current study revealed that 42.03% and 18.47% of the adult population of Zahedan suffer from pre-HTN and HTN, respectively. The World Health Organization reports the prevalence of HTN in the Eastern Mediterranean region between 14.7% and 26.4% [29]. A systematic review in the Middle East reported the prevalence of pre-HTN and HTN as 28.60% and 24.36%, respectively [30]. In line with this study, the results of other studies in different regions of Iran reported the prevalence of HTN between 17 and 22 percent [31–34]. The results of the STEPS study in Iran showed that the prevalence of HTN in Sistan and Baluchistan province is 19.90% (STEPS 2016). Two key factors may account for the observed difference: firstly, our study focused on an urban population, while STEPS encompassed both rural and urban areas. Secondly, there is an age disparity between our study population (35–70 years) and STEPS, which included individuals aged >18 years [35].

The prevalence of pre-HTN in this region was 42.03%, which was higher than the global prevalence (36%) [36]. The fact that a substantial proportion of the adult population of Zahedan has pre-HTN and HTN highlights the potential future burden of CVDs in the region [37].

The present study showed that the prevalence of pre-HTN and HTN was higher in men than women, possibly due to men’s higher prevalence of metabolic risk factors [38,39]. A study conducted in Fars province reported contradictory results [40], but most previous studies in Iran reported similar findings [20,41,42]. In this study, HTN was more common in the elderly. This pattern was consistent with other studies conducted in Iran [20,43,44]. Our results align with the findings of other Asian countries such as China, Bangladesh, and Saudi Arabia [11,45,46]. The relationship between the prevalence of HTN and aging is consistent with the overall picture in other developed and developing countries [11,47–49]. As individuals grow older, various physiological changes occur in the body, including increased arterial stiffness and changes in vascular structure, which can contribute to higher blood pressure [50,51]. In addition, older individuals are more likely to have comorbidities, such as diabetes, heart disease, and chronic kidney disease, which are closely intertwined with hypertension [52].

This study showed an inverse relationship between HTN and education level; these findings were similar to a previous study in Iran [40] and other countries [53–56]. The observed inverse relationship between education level and HTN highlights the complex interplay between socio-economic factors, health literacy, and preventive health behaviors. Education is the key factor shaping health literacy and acts as a mediator linking socioeconomic status to health outcomes, quality of life, specific health indicators, health behaviors, and preventive service utilization [57]).

Also, this study showed that people with higher SES have higher odds of having pre-HTN and HTN. The special food culture of the region, as well as the power to buy and consume more fast food in people with a higher SES level, can be a factor for overweight and obesity and ultimately increase blood pressure. The results agreed with the Indian survey, which may be due to the cultural similarity of some Indian customs with this province [58].

Consistent with the results of other studies from Iran and other parts of the world, our study showed that the odds of developing pre-HTN and HTN increase with increasing BMI and WC [34,44,59,60]. Therefore, overweight and obesity in our study were among the most important modifiable risk factors for pre-HTN and HTN. Evidence has shown that the combination of obesity and HTN may increase CVD [61], so weight control should be a priority for populations with HTN. The association between obesity and HTN can be attributed to several underlying mechanisms. Excess body fat, especially visceral fat, can lead to increased insulin resistance, inflammation, and sympathetic nervous system activity, all of which contribute to elevated blood pressure [62].

The prevalence of pre-HTN and HTN was higher in people with a history of CVD in our study. High blood pressure is the primary risk factor for CVD and is associated with increased traditional CVD risk factors. Starting at a systolic blood pressure level of 90 mm Hg, there is a 53% higher risk for atherosclerotic cardiovascular disease for each 10-mm Hg systolic blood pressure increase [63,64]. These results were consistent with other studies conducted in Iran and globally that showed that the association between HTN and a history of CVD is continuous and independent of other risk factors [5,15,40,65].

According to the results of this study, a positive history of diabetes acts as a risk factor for HTN; other studies conducted in Iran and worldwide confirm this finding [40,44,66–68]. HTN and diabetes are often coexisting conditions with shared risk factors [69]. A retrospective cohort study in Japan showed a strong association between FBS and HTN, such that an increase of 10 mg/dL of FBS over five years in non-diabetic subjects increased the risk of HTN by 42.2% [70].

In the present study, a family history of HTN is a risk factor for pre-HTN and HTN, consistent with findings in other studies conducted both in Iran and around the world [20,41,43,71–73]. A cohort study in Sri Lanka showed that people with a family history of HTN were approximately 1.4 times (parents 1.28, siblings 1.27, grandparents 1.34) more likely to develop HTN than people without a family history [73].

Some studies in line with our results have shown that low HDL and high TG levels are significantly associated with pre-HTN and HTN [38,55,74,75]. A cohort study with seven years of follow-up in Finland showed that increasing a standard deviation (SD) in serum TG levels during this period increased HTN by 1/63-fold [75]. Elevated TG levels cause endothelial dysfunction, increased arterial stiffness, and consequently HTN, but high HDL levels reduce arterial stiffness [55].

### Strengths and limitations

This study boasts several strengths, including large sample size, accurate data collection, and a community-based multi-stage cluster sampling approach. However, there are some limitations to consider. The study’s cross-sectional design restricts the ability to establish causal relationships. Furthermore, Although most of the variables in this study, including the outcome variable, were objectively measured, it’s important to acknowledge that some of the independent variables relied on self-reporting, which may introduce recall bias. Furthermore, it’s important to note that this study specifically targeted an urban population, so caution should be exercised when applying these findings to the broader population. Additionally, it’s worth highlighting that this study did not explore the association between nutritional status and hypertension, suggesting a valuable avenue for future research.

## Conclusion

This study reveals that more than half of the adult population in Zahedan was affected by pre-HTN and HTN. Predictors of pre-HTN and HTN include gender, older age, illiteracy, SES, overweight and obesity, a history of CVD and diabetes, family history of HTN, and abnormal blood lipids. To address this public health concern, educational interventions aimed at increasing awareness and public health initiatives targeting modifiable factors like weight, physical activity, and diet are essential to prevent and manage HTN. Additionally, addressing pre-HTN as a significant health issue in the region by identifying and implementing lifestyle modifications is imperative to delay the onset of HTN and CVD.

## Abbreviations

OR: Odds ratio
WHO: World Health Organization
SD: Standard deviation
